# Deciphering the Pathogenicity of the *PMS2* p.His701Gln Variant in a Family with Elderly-Onset Extracolonic Lynch Syndrome

**DOI:** 10.3390/cells15211990

**Published:** 2026-10-31

**Authors:** Erica Candela, Giovanna Forte, Matteo Iacoviello, Antonia Lucia Buonadonna, Anna Maria Valentini, Martina Lepore Signorile, Paola Sanese, Katia De Marco, Andrea Manghisi, Nicoletta Maria Tutino, Emanuele Tatò, Candida Fasano, Vittoria Disciglio, Valentina Grossi, Cristiano Simone

**Affiliations:** 1 Medical Genetics, National Institute of Gastroenterology, IRCCS “Saverio de Bellis” Research Hospital, 70013 Bari, Italy; erica.candela@irccsdebellis.it (E.C.); giovanna.forte@irccsdebellis.it (G.F.); matteo.iacoviello@irccsdebellis.it (M.I.); lucia.buonadonna@irccsdebellis.it (A.L.B.); martina.lepore@irccsdebellis.it (M.L.S.); paola.sanese@irccsdebellis.it (P.S.); katia.demarco@irccsdebellis.it (K.D.M.); andrea.manghisi@irccsdebellis.it (A.M.); nicoletta.tutino@irccsdebellis.it (N.M.T.); valentina.grossi@irccsdebellis.it (V.G.); cristiano.simone@irccsdebellis.it (C.S.); 2 Department of Molecular Medicine, University of Rome La Sapienza, 00161 Rome, Italy; 3 Histopathology Unit, National Institute of Gastroenterology, IRCCS “Saverio de Bellis” Research Hospital, 70013 Bari, Italy; am.valentini@irccsdebellis.it; 4 Medical Direction, National Institute of Gastroenterology, IRCCS “Saverio de Bellis”, Research Hospital, 70013 Bari, Italy; emanuele.tato@irccsdebellis.it; 5 Department of Precision and Regenerative Medicine and Ionian Area (DiMePRe-J), University of Bari Aldo Moro, 70124 Bari, Italy

**Keywords:** PMS2, Lynch Syndrome, autosomal dominant cancer predisposition syndrome, variants of uncertain significance (VUS), *PMS2* c.2103T>A, p.His701Gln (p.H701Q), LS-associated malignancy, LS spectrum of germline variants, late-onset small bowel adenocarcinoma

## Abstract

Missense variants in *PMS2* remain difficult to interpret in Lynch Syndrome (LS), particularly when clinical evidence is limited. We investigated the *PMS2*, NM_000535.7: c.2103T>A; p.His701Gln (p.H701Q) variant, currently classified as a variant of uncertain significance (VUS), identified in a 71-year-old patient with small intestinal adenocarcinoma. To clarify its clinical significance, we combined clinical, in silico, and functional analyses. Computational predictions consistently pointed to a deleterious effect, with AlphaMissense assigning a highly damaging score and most independent predictors supporting the same interpretation. H701Q affects the highly conserved C-terminal region of PMS2, close to the functionally critical Glu705 residue. In HEK-293T cells, PMS2-H701Q showed markedly reduced protein levels compared with wild-type PMS2, supporting an effect on protein stability. Co-immunoprecipitation experiments further showed a substantial reduction in the interaction between PMS2-H701Q and MLH1, indicating impaired MutLα complex formation. Both effects were comparable to those observed for the pathogenic PMS2-E705K control. Overall, the agreement between computational and functional findings provides compelling evidence for a deleterious effect of H701Q and strengthens its clinical interpretation. Collectively, these findings demonstrate the utility of functional evidence under the ClinGen-InSiGHT PMS2-specific ACMG/AMP variant classification guidelines and support reclassifying the *PMS2*, NM_000535.7: c.2103T>A; p.His701Gln (p.H701Q) variant from a Variant of Uncertain Significance (VUS) to likely pathogenic.

## 1. Introduction

As an evolutionarily conserved post-replicative proofreading system, the DNA mismatch repair (MMR) pathway corrects base-pairing and micro-insertion/deletion errors that escape DNA polymerase fidelity during genome duplication. This pathway is essential for maintaining eukaryotic genome integrity [1,2]. In humans, two highly conserved heterodimeric protein complexes mediate this pathway: MutS and MutL.

MutS and MutL are two highly conserved heterodimeric protein complexes that mediate this pathway in humans. MutS recognizes base–base mismatches and insertion–deletion loops (IDLs) [3]. MutL coordinates downstream degradation of the mismatch-carrying DNA strand, excision of the error-containing DNA strand, and the subsequent DNA resynthesis needed to restore genome integrity [4,5]. Etiologically, Lynch Syndrome (LS) arises from germline disease-causing variants across the MMR pathway (*MLH1*, *MSH2*, *MSH6*, *PMS2*) or via structural *EPCAM* deletions that trigger transcriptional inactivation of *MSH2* [6]. LS is associated with a markedly increased lifetime risk of colorectal and endometrial cancers, as well as an elevated risk of a characteristic spectrum of extracolonic malignancies, including tumors of the stomach, small intestine, urinary tract, ovaries, and central nervous system [7,8]. Within the MMR machinery, PMS2 acts as the endonuclease subunit of the MutLα heterodimer and confers the complex’s catalytic activity. In contrast, MLH1 mainly functions as a structural scaffold, stabilizing the complex and coordinating its interactions with other repair factors. Thus, while MLH1 provides the structural framework, PMS2 contributes the nuclease activity required for efficient DNA MMR [5]. Although *MLH1* and *MSH2* have traditionally been recognized as the main genetic determinants of LS, *PMS2* is increasingly recognized as an important, yet still less well-characterized, contributor to the LS spectrum of germline variants [6]. The relatively limited recognition of *PMS2*-associated disease is partly due to the technical challenges of analyzing this gene. In particular, the *PMS2* locus has highly similar paralogous sequences, including the pseudogene *PMS2CL*. This high degree of sequence homology can complicate variant detection and interpretation, making accurate molecular screening of *PMS2* considerably more challenging [4]. These diagnostic challenges are further complicated by the distinct clinical and molecular features associated with *PMS2* variants. Heterozygous carriers of pathogenic *PMS2* variants generally exhibit reduced cancer penetrance and a significantly later age of tumor onset compared with individuals carrying pathogenic variants in other MMR genes [9,10].

Cumulative cancer risks among *PMS2* variant carriers by age 70 are estimated at 15–20% for colorectal cancer (CRC) and 25–32% for any LS-associated malignancy. These risks are substantially lower than the approximately 70–80% lifetime cancer penetrance reported for individuals carrying pathogenic variants in *MLH1* or *MSH2* [9,11].

This difference in clinical presentation may reflect distinct biological features of *PMS2*-deficient cells [12]. In contrast to tumors arising in *MLH1* or *MSH2* carriers, which can develop through a more aggressive MMR-deficient (dMMR) crypt pathway characterized by rapid tumor progression and early-onset interval cancers, *PMS2*-associated tumors appear to more often follow the conventional adenoma-to-carcinoma sequence. In these tumors, loss of MMR function may occur later during tumor development, which could contribute to the later age at which cancer is typically diagnosed in *PMS2* carriers, often during the sixth or seventh decade of life [12].

The molecular features of *PMS2*-deficient tumors also point to a distinct pattern of tumor development. Genomic studies have shown that activating alterations in the Wnt signaling pathway, particularly *CTNNB1* mutations, are commonly found in *MLH1*-associated tumors but are rarely detected in CRC arising in *PMS2* carriers [4]. Moreover, *PMS2*-deficient tumors display a unique distribution of *KRAS* hotspot mutations compared with other MMR-deficient CRCs, suggesting that the evolutionary trajectory, timing of MMR inactivation, and selective pressures underlying *PMS2*-driven tumorigenesis differ fundamentally from those associated with other LS genes [4]. Among the extracolonic manifestations of LS, small-bowel adenocarcinoma is a rare but clinically important sentinel malignancy [13,14]. Although constitutional MMR deficiency (CMMRD), a severe childhood cancer predisposition syndrome caused by biallelic pathogenic variants in MMR genes, is frequently associated with small bowel tumors arising during the first decades of life, monoallelic LS carriers typically develop these malignancies at a substantially later age. Consequently, the occurrence of small bowel adenocarcinoma in an older adult may serve as an important clinical clue to an underlying *PMS2* germline pathogenic variant, particularly in families lacking a striking CRC history because of the reduced penetrance of *PMS2* [12].

A major challenge in the clinical management of LS remains interpreting variants of uncertain significance (VUS) [15]. Missense variants, in particular, may produce a full-length protein that is retained and detectable by immunohistochemistry (IHC) despite functional impairment [7]. This issue is especially relevant for the C-terminal endonuclease domain of PMS2, which contains the catalytic machinery required for its latent endonuclease activity. Variants affecting this region may selectively impair the protein’s ability to introduce the strand-specific nick required to initiate MMR while preserving its interaction with MLH1 and, consequently, maintaining apparently normal protein expression by IHC [4].

By taking a multidisciplinary approach, this study combines genetic, clinical, somatic, bioinformatic, and experimental methods to evaluate how the identified *PMS2* missense variant affects structural stability and the clinical phenotype associated with LS. In this study, we describe a proband investigated following the diagnosis of a small bowel adenocarcinoma in a 71-year-old proband, who also presented with a metachronous sigmoid colon adenocarcinomatous polyp. Genetic analysis identified a rare missense variant in *PMS2*, NM_000535.7: c.2103T>A; p.His701Gln (p.H701Q), classified as a variant of uncertain significance (VUS). This variant lies within the highly conserved catalytic region of the C-terminal domain, which is also part of the MLH1 interaction domain. Given the patient’s age and the specific tumor site, this case exemplifies the emerging profile of *PMS2*-associated LS as a late-onset, distinct clinical entity. By analyzing segregation of this variant within the kindred and evaluating its position within the structural framework of the MutLα complex, we assessed the potential pathogenicity of the *PMS2* c.2103T>A; p.His701Gln (p.H701Q) variant. Our findings highlight the importance of expanding clinical suspicion for LS among older patients with extracolonic tumors and support the global effort to reclassify *PMS2* VUS to improve cancer surveillance accuracy.

## 2. Materials and Methods

### 2.1. Patient Selection and Clinical Indication for Genetic Testing

The proband was evaluated during counseling at the Medical Genetics Unit of the National Institute of Gastroenterology IRCCS ‘Saverio de Bellis’ following a diagnosis of a small bowel adenocarcinoma at age 70, in conjunction with a metachronous sigmoid colon adenocarcinomatous polyp. The proband’s eligibility for germline multigene panel sequencing was based on diagnostic algorithms from the National Comprehensive Cancer Network (NCCN) Guidelines for Genetic/Familial High-Risk Assessment: Colorectal, Endometrial, Esophageal, and Gastric (Version 01.2026) [16]. Specifically, germline testing was indicated by two converging clinical and laboratory findings. First, the presence of dual primary neoplasms belonging to the Lynch spectrum (small bowel and colorectal adenocarcinoma), regardless of age at diagnosis, and the absence of PMS2 nuclear staining on immunohistochemistry (IHC), while MLH1, MSH2, and MSH6 expression remained intact.

Routine diagnostic molecular and genetic analyses were conducted on peripheral blood and/or tissue specimens [14]. The proband granted written informed consent prior to enrollment, and the study was executed in accordance with the Declaration of Helsinki under institutional ethics committee approval (Protocol code N_170; approved 31 October 2016).

### 2.2. Germline Genetic Analysis

Genomic DNA was isolated from peripheral blood using the MagCore^®^ Genomic DNA Whole Blood Kit (Amerigo Scientific, New York, NY, USA) following the manufacturer’s protocol. DNA from the family’s index cases was then sequenced and analyzed using the targeted Next Generation Sequencing (NGS) multigene panel (Ion AmpliSeq™ BRCA Reflex—Hereditary Cancer Research Panel, Thermo Fisher Scientific, Waltham, MA, USA) to examine the entire coding regions of 25 genes (*APC*, *ATM*, *BARD1*, *BMPR1A*, *BRIP1*, *CDH1*, *CDK4*, *CDKN2A*, *CHEK2*, *EPCAM*, *MLH1*, *MRE11A*, *MSH2*, *MSH6*, *MUTYH*, *NBN*, *PALB2*, *PMS2*, *PTEN*, *RAD50*, *RAD51C*, *RAD51D*, *SMAD4*, *STK11*, and *TP53*) linked to hereditary cancer, as previously described [14,17].

The classification of germline variants is guided by ClinGen-InSiGHT PMS2-specific ACMG/AMP variant classification guidelines (ClinGen; https://clinicalgenome.org/, accessed on 10 June 2026) [18]. The variants likely causing the clinical features in the index case were confirmed by Sanger sequencing. To verify the PMS2 variant, we used primers targeting unique genomic regions of *PMS2* in long-range PCR, a technique that amplifies *PMS2* while reducing interference from its homologous pseudogene [19]. We then performed exon-specific amplification using the primers and conditions previously described by Vaughn et al. [19]. Sanger sequencing was performed using the BigDye™ Terminator Cycle Sequencing Kit (Thermo Fisher Scientific, Waltham, MA, USA), with each sample analyzed on the SeqStudio™ Genetic Analyzer (Thermo Fisher Scientific, Waltham, MA, USA) [14].

### 2.3. Copy Number Analysis

The QIAamp DNA FFPE Tissue Kit (Qiagen, Hilden, Germany) was used to extract genomic DNA from formalin-fixed paraffin-embedded (FFPE) tissue slices from the index case, in accordance with the manufacturer’s instructions. The MSI status of FFPE tumor samples was evaluated using the fully automated Idylla MSI Test real-time PCR system (Biocartis, Mechelen, Belgium), in accordance with the manufacturer’s protocol and the previously described methodology [14]. Furthermore, we identified the *BRAF* V600E mutation in DNA extracted from FFPE CRC tissues using the Idylla BRAF Mutation Test (Biocartis, Mechelen, Belgium), following the manufacturer’s procedure and the previously disclosed methodology [20]. Methylation analysis of the human *MLH1* gene promoter was performed in two replicates for each tumor DNA sample using the ME011-Mismatch Repair genes kit with MS-MLPA (MRC-Holland, Amsterdam, The Netherlands), with leukocyte DNA samples as controls. The MS-MLPA procedure followed the manufacturer’s instructions and as previously described [14]. PCR products were analyzed on the SeqStudio™ Genetic Analyzer (Thermo Fisher Scientific, Waltham, MA, USA). Data analysis was performed with Coffalyser.Net software (version v.260901.1020, MRC Holland, The Netherlands).

The methylation status of the *MLH1* promoter gene was determined in accordance with the methodology outlined in a previous paper [21].

### 2.4. Immunohistochemistry Analysis

Human tissue specimens were fixed in 4% buffered formalin and embedded in paraffin. Formalin-fixed paraffin-embedded (FFPE) sample of the index case was cut into sequential sections (4 µm thick). Immunohistochemical staining was performed on a BOND III automated immunostainer (Leica Biosystems, Nussloch GmbH, Germany), covering all steps from deparaffinization to hematoxylin counterstaining, using the Bond Polymer Refine Detection Kit (DS9800, Leica Biosystems, Nussloch GmbH, Germany). After deparaffinization, the sections were rehydrated in distilled water and incubated with 3% hydrogen peroxide for 10 min to inhibit endogenous peroxidase activity. Next, the sections were incubated overnight with the following primary antibodies: FLEX Monoclonal Mouse Anti-MLH1, Clone ES05 (GA079, Agilent Dako Omnis, Santa Clara, CA, USA), FLEX Monoclonal Mouse Anti-MSH2, Clone FE11 (GA085, Agilent Dako Omnis, Santa Clara, CA, USA), FLEX Monoclonal Rabbit Anti-MSH6, Clone EP49 (GA086, Agilent Dako Omnis, Santa Clara, CA, USA), and FLEX Monoclonal Rabbit Anti-PMS2, Clone EP51 (GA087, Agilent Dako Omnis, Santa Clara, CA, USA). The EnVision FLEX Target Solution, High pH (50×), was used for antigen retrieval (GV804, Agilent Dako Omnis, Santa Clara, CA, USA). Images were captured with a Zeiss Axio Observer Z1 optical microscope (Carl Zeiss, Oberkochen, Germany). The analyzed MMR proteins were considered to have a normal expression pattern when they stained positively in neoplastic cells. Conversely, a lack of nuclear staining, despite positive internal controls (such as normal mucosa, stromal cells, endothelial cells, or lymphocytes), was regarded as a defect in protein expression.

### 2.5. In Silico Prediction Analysis

To evaluate the evolutionary conservation and potential functional impact of the missense substitution, we aligned human PMS2 protein sequences with orthologous sequences across diverse species using Align-GVGD (https://maialab.org/agvgd/; accessed on 18 May 2026 [22]). As previously reported, this algorithm integrates multiple sequence alignments with amino acid biophysical properties to stratify variants along a spectrum from benign to severely damaging [14].

To examine the structural and functional consequences of the VUS (c.2103T>A; p.His701Gln) found in the *PMS2* gene, we evaluated its impact on the human PMS2 protein’s molecular structure. This assessment, based on an in silico prediction meta-analysis, utilized eighteen computational tools, including AlphaMissense (https://alphamissense.hegelab.org/; accessed on 18 May 2026 [23]), CADD (https://cadd.gs.washington.edu/info; accessed on 18 May 2026 [24]), ClinPred (https://sites.google.com/site/clinpred/home?authuser=0; accessed on 18 May 2026 [25]), DEOGEN 2 (https://www.dbnsfp.org/; accessed on 18 May 2026 [26]), E-SNP&GO (https://esnpsandgo.biocomp.unibo.it/; accessed on 18 May 2026 [27]), EVE (https://evemodel.org; accessed on 18 May 2026 [28]), FATHMM (http://fathmm.biocompute.org.uk/; accessed on 18 May 2026 [29]), LIST-S2 (https://list-s2.msl.ubc.ca/; accessed on 18 May 2026 [30]), MetalR (https://www.ensembl.org/Tools/VEP; accessed on 18 May 2026 [31]), MetaSVM (https://github.com/jjh0925/metaSVM, accessed on 18 May 2026 [31]), MutPred2 (http://mutpred.mutdb.org/ [32]), Mutation Taster (www.mutationtaster.org; accessed on 18 May 2026 [33]), Mutation Assessor (http://mutationassessor.org/r3/; accessed on 18 May 2026 [34]), PANTHER (http://www.pantherdb.org; accessed on 18 May 2026 [35]), PolyPhen-2 (https://genetics.bwh.harvard.edu/pph2/, accessed on 15 June 2026 [36]), PROVEAN (http://provean.jcvi.org [37]), REVEL (https://sites.google.com/site/revelgenomics, accessed on 15 June 2026 [38]), SIFT (https://siftdna.org/ [39]). These tools used different approaches and methodologies and did not require the full PDB structure of PMS2 as input, which has not yet been defined.

Among eighteen tools, we used AlphaMissense, an AI model derived from AlphaFold v2.3.2, the only software that provides a prediction of the structure of PMS2. It predicts the pathogenicity of missense variants by integrating structural context with amino acid evolutionary conservation. The algorithm generates a pathogenicity score for each missense variant based on multiple genetic and experimental factors [23].

Another in silico prediction tool in our meta-analysis is PolyPhen 2 (Polymorphism Phenotyping v2). It assesses both sequence-based and structure-based features, comparing the wild-type (ancestral, normal) allele with the mutant (derived, disease-causing) allele to determine the effect of an amino acid substitution [36]. For instance, PROVEAN is a tool that calculates sequence alignment scores and generates precomputed predictions for 20 possible single-amino-acid substitutions and one deletion at each amino acid position across all human and mouse protein sequences [14,40].

### 2.6. Cell Lines and Cell Transfection

The HEK-293T cell line (ATCC^®^ CRL-3216™) was obtained from ATCC (Manassas, VA, USA) and cultured in DMEM high glucose without pyruvate (catalog 41965-039, Gibco, Grand Island, NY, USA), supplemented with 10% FBS (A5256701, Gibco, Grand Island, NY, USA), 100 IU/mL penicillin-streptomycin (15140122, Gibco, Grand Island, NY, USA), 1% sodium pyruvate (11360-039, Gibco, Grand Island, NY, USA), and 1% MEM non-essential amino acids (NEAA) (11140-035, Gibco, Grand Island, NY, USA). Cultivation took place at 37 °C in a 5% CO_2_ incubator, with regular testing for mycoplasma contamination (117048, Minerva Biolabs, Berling, Germany) as previously described [41]. HEK-293T cells were transiently transfected with mammalian expression plasmids using Lipofectamine 3000 (L3000008, Thermo Fisher Scientific, Waltham, MA, USA) following the manufacturer’s protocol.

### 2.7. Cloning and Mutagenesis

The plasmids described in this manuscript were generated using specific primers, as previously described [14]. In this manuscript, we utilized the (#16475, Addgene, Watertown, MA, USA), pEGFP-N1 (#6085-1, Clontech, Mountain View, CA, USA), and pcDNA3.1 empty (V79020, Thermo Fisher Scientific, USA) plasmids. Dr. Guido Plotz kindly provided the pcDNA3.1_hMLH1 and pSG5_PMS2-WT vectors. The pSG5_hPMS2_H701Q and pSG5_hPMS2_E705K constructs were generated by site-directed mutagenesis, performed using the Q5 Site-Directed Mutagenesis Kit (E0554S, New England Biolabs, Ipswich, MA, USA) according to the manufacturer’s instructions and as previously described [41]. Site-directed mutagenesis was performed using the following primers:
SDM PMS2 H701Q FWAGTGGACCAGgaaGCCACGGACGSDM PMS2 H701Q RVATGAAGATATCCTCATTCAGTTTGGTTATTATAAATCSDM PMS2 Q705K FWTGCCACGGACctgAAGTATAACTTCGAGSDM PMS2 Q705K RVTGCTGGTCCACTATGAAGATATC

Efficiency DH5α competent cells (C2987H, New England Biolabs, Ipswich, MA, USA) were purchased from New England Biolabs and were used for all cloning experiments performed in this study. Cells were grown in Luria broth medium (10855021, Gibco, Grand Island, NY, USA) [41].

### 2.8. Immunoblotting

Cells were harvested and lysed in buffer (50 mM Tris-HCl pH 7.4, 5 mM EDTA, 250 mM NaCl, and 1% Triton X-100) with added protease and phosphatase inhibitors (78443, Thermo Fisher Scientific, Waltham, MA, USA) [41]. Each sample’s 40 μg of protein was denatured in 4× Laemmli buffer (1610747, Bio-Rad Laboratories, Hercules, CA, USA), loaded onto an SDS-PAGE gel, and transferred to a 0.2 μm nitrocellulose membrane. The membranes were blocked with PBS containing 1% casein (#1610783, Bio-Rad Laboratories, Hercules, CA, USA) and incubated overnight at 4 °C with primary antibodies: anti-β-actin (4970, Cell Signaling Technology, Danvers, MA, USA), anti-GFP (2956, Cell Signaling Technology, Danvers, MA, USA), anti-MLH1 Clone ES05 (GA079, Agilent Dako, Santa Clara, CA, USA), and anti-PMS2 Clone EP51 (GA087, Agilent Dako, USA). Secondary detection used Rabbit IgG HRP and Mouse IgG HRP (1706515 and 1706516, Bio-Rad Laboratories, Hercules, CA, USA), visualized with Clarity Western ECL Substrate (1705061, Bio-Rad Laboratories, Hercules, CA, USA).

Densitometric analysis of the Western blot was performed by first normalizing MLH1 and PMS2 expression levels to GFP to assess transfection efficiency, and subsequently to the loading control (β-actin). Fold Changes relative to the MLH1 + PMS2 WT sample were determined separately for three independent experiments (Experiments 1–3) as reported in Supplementary Material.

### 2.9. Co-Immunoprecipitation

Cells were collected and homogenized in lysis buffer (50 mM Tris-HCl pH 7.4, 5 mM EDTA, 250 mM NaCl, and 1% Triton X-100) supplemented with protease and phosphatase inhibitors (78443, Thermo Fisher Scientific, Waltham, MA, USA) [41]. Coupling between Dynabeads Protein G (10003D, Thermo Fisher Scientific, Waltham, MA, USA) and the anti-MLH1 Clone ES05 antibody (GA079, Agilent Dako, Santa Clara, CA, USA) was performed in 100 μL of 0.01% Tween-20 in 1X PBS for 45 min at room temperature on a rocking platform. Cell lysates were immunoprecipitated with antibody-bead complexes [41]. Immunocomplexes were washed extensively, boiled in 2× Laemmli sample buffer (1610737, Bio-Rad Laboratories), and subjected to SDS-PAGE and immunoblot analysis. Input corresponds to 5% of the lysate. IgG was used as a negative control [41]. Primary antibodies: anti-MLH1 Clone ES05 (GA079, Agilent Dako, Santa Clara, CA, USA), and anti-PMS2 Clone EP51 (GA087, Agilent Dako, Santa Clara, CA, USA). Secondary detection used Rabbit IgG HRP and Mouse IgG HRP (1706515 and 1706516, Bio-Rad Laboratories, Hercules, CA, USA), visualized with Clarity Western ECL Substrate (1705061, Bio-Rad Laboratories, Hercules, CA, USA).

Densitometric quantification of MLH1 co-immunoprecipitation (IP-MLH1) assays was performed by normalizing the PMS2 (prey) signal intensity to MLH1 (bait) (PMS2/MLH1 ratios), and Fold Changes relative to the MLH1 + PMS2 WT co-immunoprecipitated sample were determined separately for three independent experiments (Experiments 1–3) as reported in Supplementary Material.

### 2.10. Statistical Analysis

Two-tailed one-sample Student’s *t*-test was used to compare the Fold Change values of each sample from three independent experiments (Experiments 1–3) with the reference value of 1 from the MLH1 + PMS2 WT sample in both Western blot and co-immunoprecipitation assays.

## 3. Results

### 3.1. Clinical History and Genetic Findings

**Figure 1:**
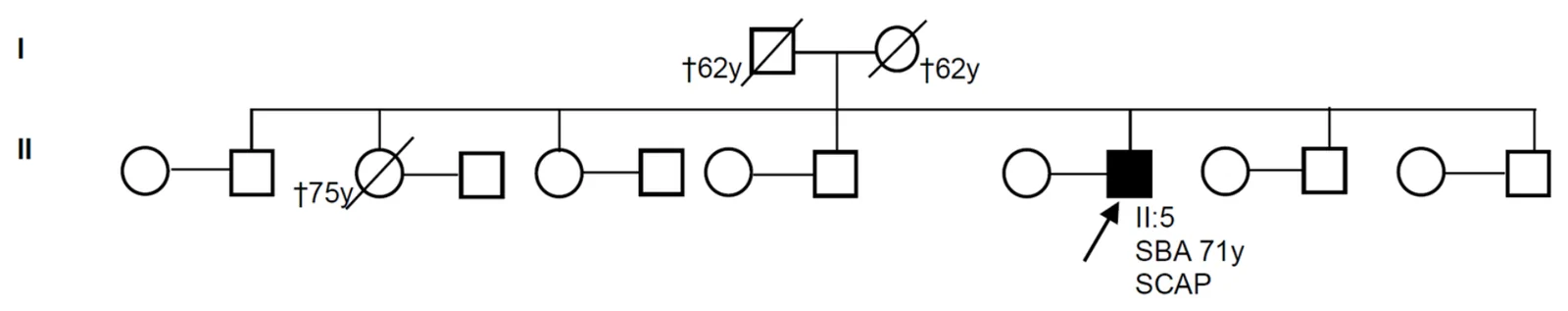
Pedigree of the family involved in this study. Squares indicate men and circles indicate women. The arrow indicates the index case. Black-filled symbols denote individuals with cancer, and unfilled symbols indicate unaffected individuals. Slashed symbols denote dead individuals. The following clinical manifestations are noted below each filled symbol: Small bowel adenocarcinoma (SBA); Sigmoid colon adenocarcinomatous polyp (SCAP); age at diagnosis (y = years); age at death (†).

We report the case of a 71-year-old man with metachronous gastrointestinal neoplasms: a sigmoid colon adenocarcinomatous polyp and an adenocarcinoma of the small intestine. The lesions were diagnosed five months apart. The index case (Figure 1, II:5) was a 71-year-old male referred to the Department of Surgery at our Institute. He was the fifth of seven siblings, all reported to be in good health, while both parents were deceased at the time of genetic counseling due to non-oncological causes.

Given the presence of metachronous gastrointestinal lesions and the results of immunohistochemical (IHC) analysis performed on FFPE tissue from the small intestine lesion, as discussed below, germline genetic testing using a panel of genes associated with hereditary predisposition to gastrointestinal cancer was performed because of the suspicion of LS.

**Figure 2:**
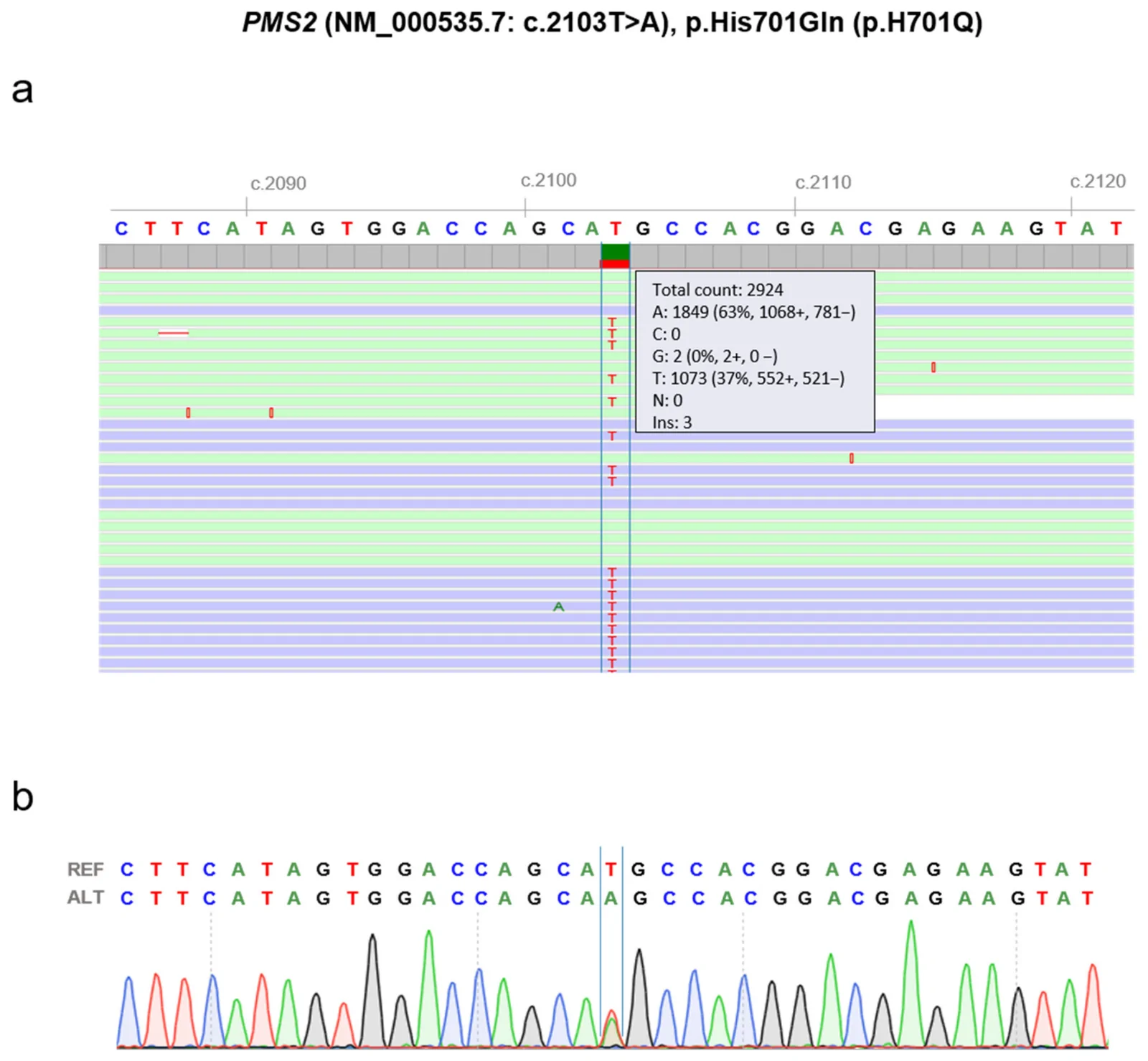
Molecular Analysis of the *PMS2* gene. Sequence analysis showing a rare missense variant in *PMS2* (NM_000535.7: c.2103T>A), p.His701Gln (p.H701Q), detected in the index case. (**a**) Next-generation sequencing (NGS) read alignment showing the *PMS2* (NM_000535.7: c.2103T>A) variant in the index case. The vertical light blue lines mark the variant position, a single-nucleotide substitution of T with A; the allele counts at this position are reported in the inset box. (**b**) Sanger sequencing electropherogram of genomic DNA from the index case confirming the *PMS2* (NM_000535.7: c.2103T>A) variant, indicated by the vertical light blue lines. The *PMS2* reference sequence (REF) and the altered sequence (ALT) are shown above the sequencing electropherogram.

This analysis identified a variant in the *PMS2* gene (NM_000535.7: c.2103T>A; p.His701Gln). The identified *PMS2* missense variant has not been described in major clinical and genetic databases (ClinVar, https://www.ncbi.nlm.nih.gov/clinvar/, accessed on 19 October 2026; InSiGHT, https://www.insight-database.org/, accessed on 19 October 2026). Based on this initial evidence, the variant was classified as a variant of uncertain significance (VUS) according to the ClinGen-InSiGHT *PMS2*-specific ACMG/AMP variant classification guidelines (https://cspec.genome.network/cspec/ui/svi/doc/GN139, accessed on 10 September 2026), with the following criteria applied: PS2 Supporting, PM2 Supporting, PP3 Supporting and PP4 Supporting. Sanger sequencing confirmed the variant (Figure 2a,b).

Heterozygous *PMS2* pathogenic variants are associated with attenuated cumulative lifetime risk and delayed clinical manifestation, often emerging in the seventh decade, which causes many carrier families to fall outside traditional clinical screening criteria [4,42]. Indeed, evaluation of the two-generation pedigree did not reveal any gastrointestinal and/or endometrial malignancies among family members, reflecting the characteristically reduced penetrance and delayed onset associated with *PMS2*-related LS. However, as it was not possible to establish whether the identified variant arose de novo, this possibility cannot be excluded as a potential explanation for the unremarkable family history.

Given the concordance between the clinical phenotype and the gene involved, as well as the variant location within the interaction domain between PMS2 and MLH1, its classification as a VUS seemed limiting. Therefore, we performed further analyses at different levels to reassess its classification.

### 3.2. MMR Protein Expression in the Index Case with an LS-Associated Small Bowel Adenocarcinoma

**Figure 3:**
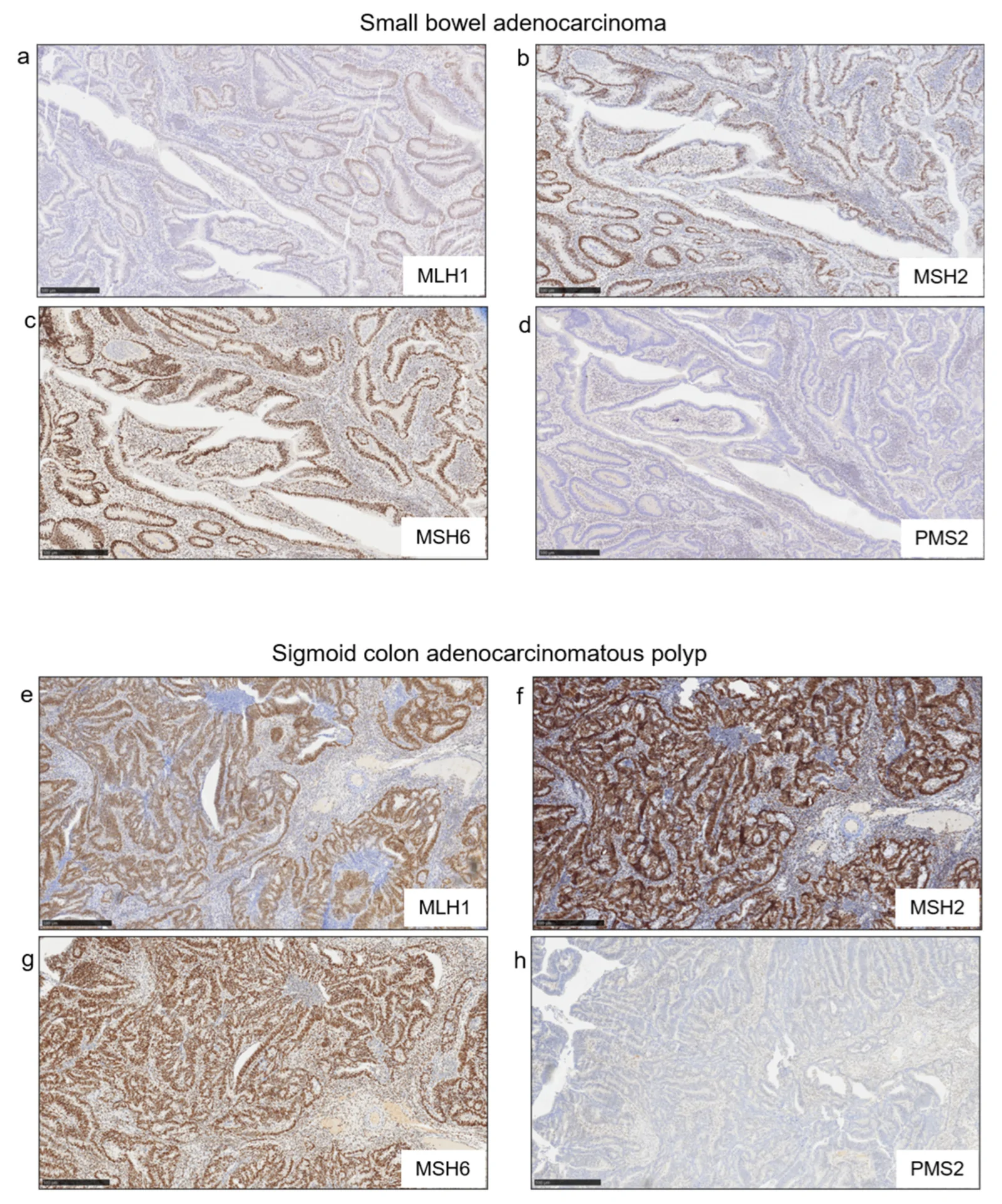
Immunohistochemical analysis of MMR proteins. Loss of PMS2 expression in index case sample tissues. (**a**–**d**) Immunohistochemical staining showing loss of PMS2 expression in the index case small bowel adenocarcinoma. Immunohistochemical analysis of mismatch repair (MMR) genes was performed on a small bowel adenocarcinoma tissue specimen from the index case (magnification, 10×). (**a**) Positive for MLH1, (**b**) positive for MSH2, (**c**) positive for MSH6 and (**d**) negative for PMS2. (**e**–**h**) Immunohistochemical analysis of mismatch repair (MMR) genes was performed on a sigmoid colon adenocarcinomatous polyp tissue specimen from the index case (magnification, 10×). (**e**) Positive for MLH1, (**f**) positive for MSH2, (**g**) positive for MSH6 and (**h**) loss of PMS2 expression.

Assessment of microsatellite instability (MSI) of the small bowel adenocarcinoma sample and sigmoid colon adenocarcinomatous polyp from the proband revealed high microsatellite instability (MSI-H). To investigate the relationship between the MSI-H phenotype and MMR protein expression, immunohistochemical analyses were performed for MLH1, MSH2, MSH6, and PMS2 on normal and cancer tissues from the same individual. The results showed the lack of PMS2 expression, while MLH1, MSH2, and MSH6 showed normal levels in small bowel adenocarcinoma (Figure 3a–d) as well as in the sigmoid colon adenocarcinomatous polyp (Figure 3e–h). Both lesions displayed a uniform, complete loss of PMS2 nuclear staining. In contrast, MLH1 nuclear expression was preserved across both lesions.

### 3.3. Bioinformatic Investigation of the Potential Functional Consequences of PMS2 p.His701Gln

Because the VUS His701Gln lies in the MLH1-binding domain at the C-terminus of PMS2, we evaluated, in silico, the structural and functional impact of the His701Gln missense in PMS2. To investigate the evolutionary conservation and potential functional relevance of this position, we compared the human PMS2 sequence with homologous proteins from different taxa, including Trichoplax adhaerens (Figure S1).

**Figure 4:**
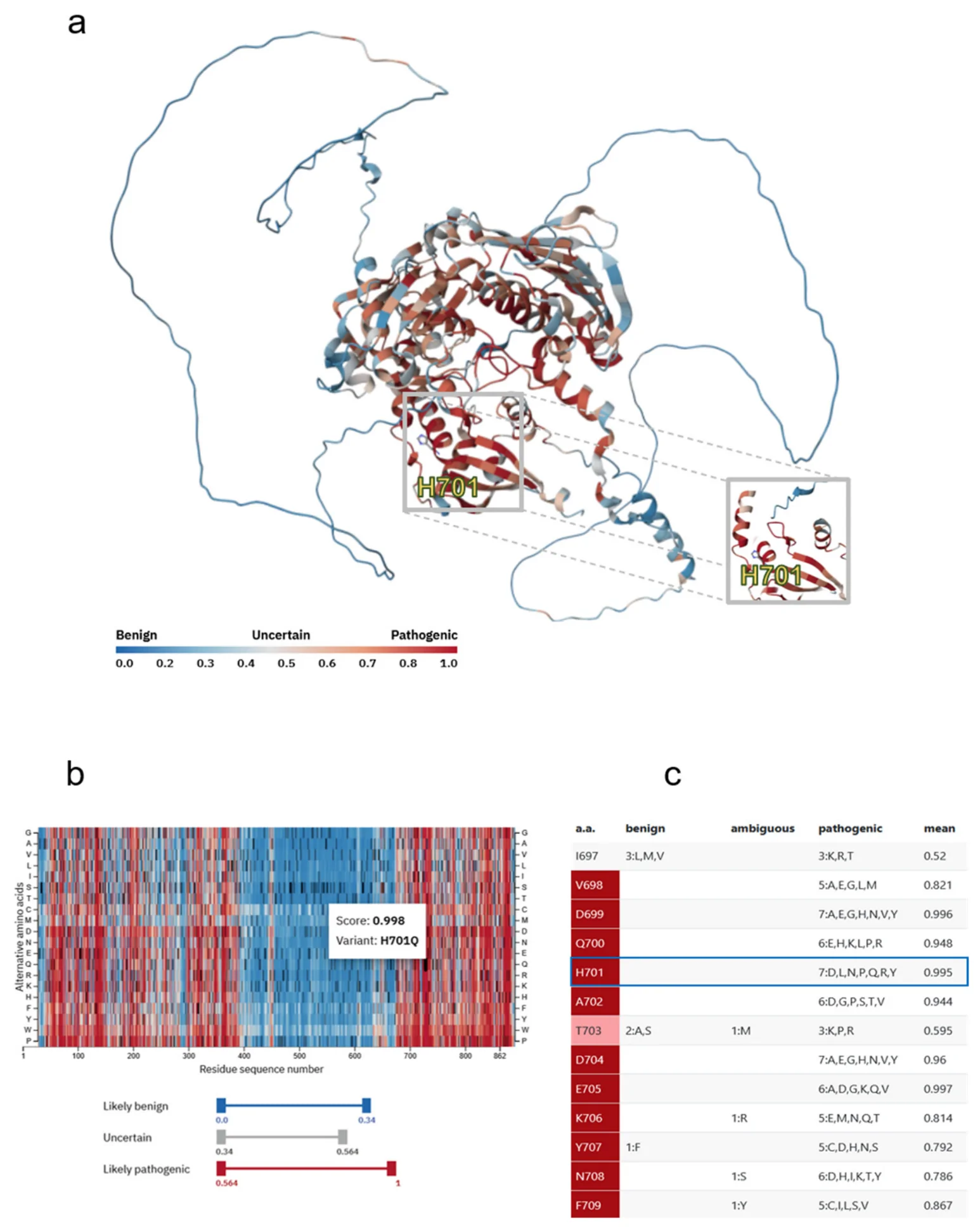
AlphaMissense-based analysis of the PMS2 p.His701Gln variant. (**a**) Three-dimensional structural representation of the PMS2 protein showing the localization of the p.His701Gln variant (H701Q) within the PMS2 domain involved in MLH1 interaction. The protein structure is colored according to AlphaMissense pathogenicity scores, ranging from benign (blue) to pathogenic (red). The magnified views highlight His701 within the local structural context. (**b**) Missense heatmap showing the distribution of predicted pathogenicity scores across the PMS2 protein sequence. The p.His701Gln variant (H701Q) showed a high pathogenicity score (0.998), consistent with a potentially damaging effect on protein function. (**c**) In silico analysis of neighboring residues surrounding His701 (amino acids 697–709) that displayed high pathogenicity scores.

Next, we investigated the potential structural impact of the identified PMS2 variant through in silico approaches. AlphaMissense analysis predicted a deleterious effect for the His701Gln substitution, with a pathogenicity score of 0.998 (Figure 4a,b). Notably, the PMS2 region encompassing His701 exhibits high AlphaFold prediction confidence, as indicated by elevated pLDDT values, supporting the reliability of structure-based interpretations at this site. Therefore, the high-confidence structural context of the His701Gln variant strengthens the robustness and interpretability of the predicted pathogenic effect (Figure 4a–c). Notably, AlphaMissense analysis showed that residues flanking His701 (amino acids 698–709) exhibited consistently high pathogenicity scores, approaching 1, indicating strong intolerance to amino acid substitutions in this region. The marked conservation and predicted deleteriousness of missense changes across this segment, located within the PMS2 MLH1-binding domain, underscore its structural and functional relevance and further support the potential pathogenic impact of the p.His701Gln variant (Figure 4c). Subsequently, to further assess the potential functional relevance of the PMS2 variant, we performed an in silico meta-analysis combining predictions from 18 computational tools. The His701Gln substitution showed a strong pathogenicity consensus, with 16/18 predictors classifying the variant as damaging (Consensus Binary Score = 0.889; 88.9%) (Table S1). This score, calculated as the proportion of damaging predictions among all included predictors, supports the predicted deleterious effect of the variant.

### 3.4. PMS2 His701Gln Impairs PMS2 Stability and MutLα Complex Formation

**Figure 5:**
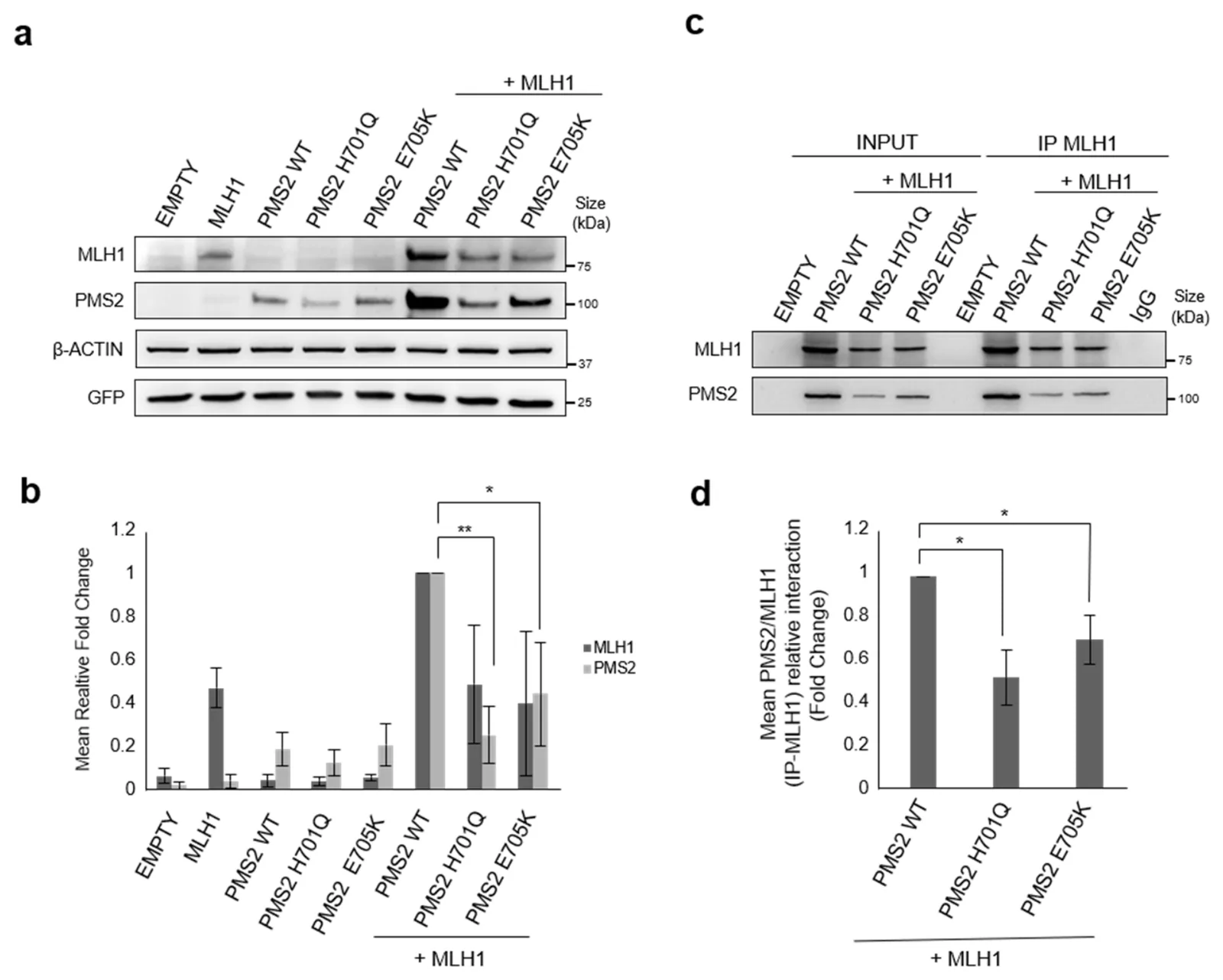
PMS2 stability and MLH1 interaction. (**a**) Immunoblot analysis of PMS2 and MLH1 in HEK-293T cells transiently transfected for 48 h with pEGFP-N1 and pcDNA3.1 EMPTY or pcDNA3.1_hMLH1 wild-type (WT), and either pSG5_hPMS2-WT or pSG5_hPMS2_H701Q or pSG5_hPMS2_E705K. β-actin was used as a loading control, and GFP served as a transfection control. Data are representative of three independent experiments. (**b**) Graphical representation of mean Fold Change of PMS2 and MLH1 levels relative to the MLH1 + PMS2 WT sample, determined from three independent experiments (Experiments 1–3). Densitometric analysis was performed by first normalizing MLH1 and PMS2 expression levels to GFP to assess transfection efficiency, and subsequently to the loading control (β-actin). Statistically significant differences are indicated as follows: * *p* < 0.05 and ** *p* < 0.01. (**c**) Co-immunoprecipitation analysis of the MutLα complex in HEK-293T cells transiently transfected for 48 h, as reported above. Immunoprecipitation was performed using an anti-MLH1 antibody, and IgG was used as a negative control. Data are representative of three independent experiments. (**d**) Graphical representation of PMS2/MLH1 (IP-MLH1) mean Fold Changes relative to the MLH1 + PMS2 WT sample, determined from three independent experiments (Experiments 1–3). Densitometric analysis was performed by normalizing the PMS2 (prey) signal intensity to MLH1 (bait). Statistically significant differences are indicated with * *p* < 0.05.

To validate our working hypothesis that the VUS His701Gln impairs PMS2 protein stability, we employed HEK-293T cells, which are characterized by the silencing of both *MLH1* and *PMS2* [43]. This genetic background allowed us to assess the specific contribution of the VUS His701Gln to PMS2 stability without interference from endogenous *PMS2* wild-type (WT) expression. HEK-293T cells were transiently transfected for 48 h with mammalian expression plasmids encoding pEGFP-N1 (as a transfection control), pcDNA3.1_hMLH1-WT, pSG5_hPMS2-WT, pSG5_hPMS2_H701Q, and pSG5_hPMS2_E705K (as a positive control) mutants, alone or in combination. Immunoblot analyses showed that overexpression of the PMS2 WT plasmid markedly stabilized PMS2 protein levels. In contrast, overexpressing the PMS2_H701Q plasmid decreased PMS2 stability, consistent with our hypothesis and comparable to the positive control, PMS2_E705K (Figure 5, Figures S2 and S3, Tables S2–S5). Compared with PMS2 WT, the H701Q variant showed a significant reduction in protein abundance, consistent with impaired protein stability. Importantly, the reduction in PMS2-H701Q levels was comparable to, and in some conditions more pronounced than, that observed for the pathogenic-control variant PMS2-E705K. β-actin and GFP levels confirmed comparable protein loading and transfection efficiency, respectively (Figure 5a,b and Figure S2, Tables S2 and S3). We next investigated whether the H701Q substitution affected the ability of PMS2 to associate with MLH1 and thereby form the MutLα complex. Co-immunoprecipitation experiments using an anti-MLH1 antibody showed a clear association between PMS2 WT and MLH1, whereas the PMS2-H701Q variant showed a significant decrease and was barely detectable in the MLH1 immunoprecipitate (Figure 5c,d and Figure S3, Tables S4 and S5).

A similarly impaired association was observed for PMS2-E705K, supporting a functional defect of this variant in MutLα complex formation. Notably, the E705K control has not previously been functionally characterized for its ability to form the MutLα complex. Together, these findings indicate that the His701Gln substitution substantially compromises PMS2 stability and its interaction with MLH1, providing functional evidence for a defective MutLα complex.

## 4. Discussion

In this study, we combined in silico analyses, cellular experiments, and investigations of patient-derived material to characterize the *PMS2,* NM_000535.7: c.2103T>A; p.His701Gln (p.H701Q) variant. This integrated approach provided convergent evidence of a deleterious effect of the variant and also illustrates the difficulties that can arise when interpreting missense variants in *PMS2*, a gene whose analysis has historically been complicated by highly homologous pseudogenes [44]. We identified the p.His701Gln variant in a 71-year-old patient with small intestinal adenocarcinoma. Although pathogenic *PMS2* variants are generally associated with lower penetrance and a later age at cancer onset than pathogenic variants in *MLH1* or *MSH2*, the clinical presentation observed in our patient is consistent with the attenuated, but clinically relevant, cancer susceptibility associated with *PMS2* [15,44]. Our experimental data provide several lines of evidence supporting the functional relevance of H701Q. The in silico analyses already pointed to a deleterious effect, with AlphaMissense assigning a highly damaging score and residues surrounding His701 in the 698–709 region showing similarly high pathogenicity scores. Cellular experiments supported these predictions, showing a clear reduction in PMS2-H701Q protein levels relative to PMS2 WT. Of note, the decrease in PMS2 abundance was at least comparable to that observed for PMS2-E705K, which was included as a pathogenic reference variant affecting the same C-terminal region (Figure 5a,b and Figure S2, Tables S2 and S3). The location of H701Q within the C-terminal region of PMS2 provides a plausible structural basis for these findings. This region contains both the MLH1-interaction domain and the latent endonuclease domain of PMS2, which is required for MutLα-dependent incision during mismatch repair. It contains a metal-binding motif (DQHAX2EX4E) that is crucial for DNA incision and initiating DNA correction and resynthesis [45,46]. Notably, a previous study reported that several variants in this motif, including PMS2 H701Q, impair MMR activity [47].

The effect of H701Q was not limited to protein abundance. Co-immunoprecipitation experiments showed a marked reduction in the association between PMS2-H701Q and MLH1, indicating that the variant also affects MutLα complex formation (Figure 5c,d and Figure S3, Tables S4 and S5). In contrast, PMS2 WT associated efficiently with MLH1 under the same experimental conditions. Interestingly, PMS2-E705K showed a similarly impaired interaction, providing a useful functional comparison with a variant already known to affect PMS2 function. Although E705K has been extensively studied as a catalytically inactive PMS2 variant, its effect on PMS2–MLH1 complex formation has been less well characterized. This finding suggests that the H701Q substitution can compromise PMS2 protein stability, thereby reducing the amount of PMS2 available to participate in MMR. In addition, our data provide additional information on the functional consequences of E705K while, more importantly, showing that H701Q affects a second essential property of PMS2: its ability to form the MLH1-containing MutLα complex.

Studies using E705K as a control show that, although the protein can bind DNA, it cannot generate the nicks needed for mismatch repair [46]. Since residue 701 is part of the same critical structure, changing histidine to glutamine, even if it does not fully destabilize the protein, is likely to affect the catalytic site’s accuracy or its interaction with PCNA, which is essential for activating the endonuclease activity [48,49]. This is similar to other C-terminal VUS, such as p.Asp699His, which are in the zinc-binding motif and impair MMR function despite stable protein expression [44,45].

These observations emphasize the importance of integrating functional evidence with clinical and molecular data to improve *PMS2* variant interpretation and optimize genetic counseling and clinical management for affected individuals.

While the cumulative risk of any LS-associated malignancy is estimated at 70–80% for *MLH1*/*MSH2* variant carriers, it is estimated at only 25–32% for *PMS2* variant carriers by age 70 [44]. This late-onset presentation, exemplified by our 71-year-old proband, likely reflects functional redundancy within the MutL complex [44]. When PMS2 is deficient, its scaffold partner, MLH1, may partially preserve MMR activity by pairing with alternative partners, such as MLH3 or PMS1 [44]. Consequently, tumorigenesis in *PMS2* carriers often bypasses the aggressive “dMMR crypt” pathway observed in MLH1 families, instead following a more protracted adenoma-to-carcinoma progression [4,42]. Furthermore, while colorectal and endometrial cancers are the primary manifestations, small bowel adenocarcinoma remains a critical, albeit rarer, component of the *PMS2* spectrum [44].

In constitutional mismatch repair deficiency (CMMRD), small bowel tumors typically appear in the second decade of life. Our study indicates that in monoallelic carriers, these extracolonic tumors can act as sentinel malignancies even in their seventies. This underscores the importance of heightened clinical vigilance in older patients presenting with MSI-H extracolonic tumors [42].

A key challenge in molecular diagnosis of *PMS2*-related LS is the misleading nature of IHC. Missense variants of *PMS2* often produce a full-length protein that still retains its antigenic properties, resulting in “intact” tumor staining despite a complete loss of enzymatic activity [50]. Recent research shows that unexpected MMR protein expression occurs in up to 44% of LS cases caused by missense mutations, compared with only 11% in those caused by truncating mutations. This phenomenon of “unexpected reactivity” can cause false-negative screening results if IHC is used alone [51].

Our study underscores the need for integrated functional assays. For *PMS2*, where clinical data (such as segregation and family history) are often limited by low penetrance, functional assays are essential to move variants from “uncertain” to “likely pathogenic” [45,50].

These findings are particularly relevant for the clinical interpretation of *PMS2* variants. According to the ACMG/AMP and ClinGen-InSiGHT frameworks, the PS3 criterion is intended to capture well-established functional evidence showing an abnormal effect of a variant on gene or protein function [15,52]. In our study, two independent experimental readouts indicate a functional defect in PMS2-H701Q: reduced protein stability and impaired incorporation into the MLH1-containing MutLα complex. Using PMS2 WT and the E705K variant as experimental controls further supports this interpretation. Taken together, these results provide functional evidence of a deleterious effect of H701Q and support applying the ACMG/AMP and ClinGen-InSiGHT PS3 criterion [15,52].

Overall, the quantitative functional metrics, such as the complete in vitro MMR activity (CIMRA) assay and cell-based stability assays, provide calibrated Odds of Pathogenicity (OddsPath) that can be formally incorporated as strong functional evidence (PS3) into the ACMG/AMP variant classification criteria for *PMS2* [50].

According to the ClinGen-InSiGHT *PMS2*-specific ACMG/AMP variant classification criteria guidelines (https://cspec.genome.network/cspec/ui/svi/doc/GN139, accessed on 10 September 2026), the *PMS2* germline variant (NM_000535.7: c.2103T>A; p.His701Gln) can be reclassified from VUS (PS2 Supporting, PM2 Supporting, PP3 Supporting, PP4 Supporting) to likely pathogenic (PS2 Supporting, PS3 Moderate, PM2 Supporting, PP3 Supporting, PP4 Supporting).

A balanced interpretation of our findings requires acknowledging both the strengths and limitations of this work. Among its principal strengths, we performed a multi-tiered investigation combining long-range PCR/NGS sequencing, detailed pedigree segregation analysis, a comprehensive 18-tool in silico meta-analysis, and direct cellular functional assays evaluating both protein stability and MutLα complex formation alongside the established pathogenic control PMS2-E705K. However, certain limitations remain. First, as a single-family study, clinical co-segregation data are inherently limited by the characteristic low penetrance and late age of onset associated with *PMS2* germline variants. Second, while our functional data showed severe protein destabilization and disrupted MLH1 binding (supporting the ACMG/AMP PS3 criterion at a moderate level), we did not directly perform an in vitro reconstituted nuclease assay. Consequently, while our convergent data strongly justify reclassifying PMS2 p.His701Gln from a VUS to ‘likely pathogenic’ (Class 4) status under ClinGen-InSiGHT criteria, definitive designation as Class 5 pathogenic will require additional population-level case–control studies.

## 5. Conclusions

This case highlights the clinical relevance of identifying LS in older adults and the importance of variant reclassification when clinical and molecular findings are suggestive. The reclassification of the PMS2 p.H701Q variant as likely pathogenic, based on ACMG/AMP criteria and supported by structural modeling, underscores the value of integrating clinical, genetic, and in silico evidence to refine variant interpretation and inform clinical assessment.

## Data Availability

The original data presented in this study are openly available in FigShare at https://doi.org/10.6084/m9.figshare.33611161.

## Supplementary Materials

The following supporting information can be downloaded at: https://www.mdpi.com/article/10.3390/cells15211990/s1, Figure S1: Evolutionary conservation of the PMS2 His701 residue; Figure S2. Western blot analysis from three independent experiments related to Figure 5a,b; Figure S3. Co-IP analysis from three independent experiments related to Figure 5c,d; Table S1: In silico meta-analysis prediction; Table S2. Densitometric analysis related to Figure 5b; Table S3. Statistical analysis of fold-change values for Figure 5b; Table S4. Densitometric analysis related to Figure 5d; Table S5. Statistical analysis of fold-change values for Figure 5d.

## Abbreviations

The following abbreviations are used in this manuscript:

MMR	DNA mismatch repair
dMMR	MMR-deficient
IDLs	Insertion–deletion loops
LS	Lynch Syndrome
CMMRD	Constitutional mismatch repair deficiency
VUS	Variants of uncertain significance
IHC	Immunohistochemistry
NCCN	National Comprehensive Cancer Network
MSI-H	High microsatellite instability
WT	Wild-type
FFPE	Formalin-fixed, paraffin-embedded
