# Supplementary material for: Deciphering the Pathogenicity of the PMS2 p.His701Gln Variant in a Family with Elderly-Onset Extracolonic Lynch Syndrome
Source: Cells. 2026 Oct 31;15(21):1990. doi: 10.3390/cells15211990 (PMC13762171; doi:10.3390/cells15211990)
Supplement: Supplementary file 1 [file cells-15-01990-s001.zip › cells-4616046-supplementary final.pdf]

Figure S1

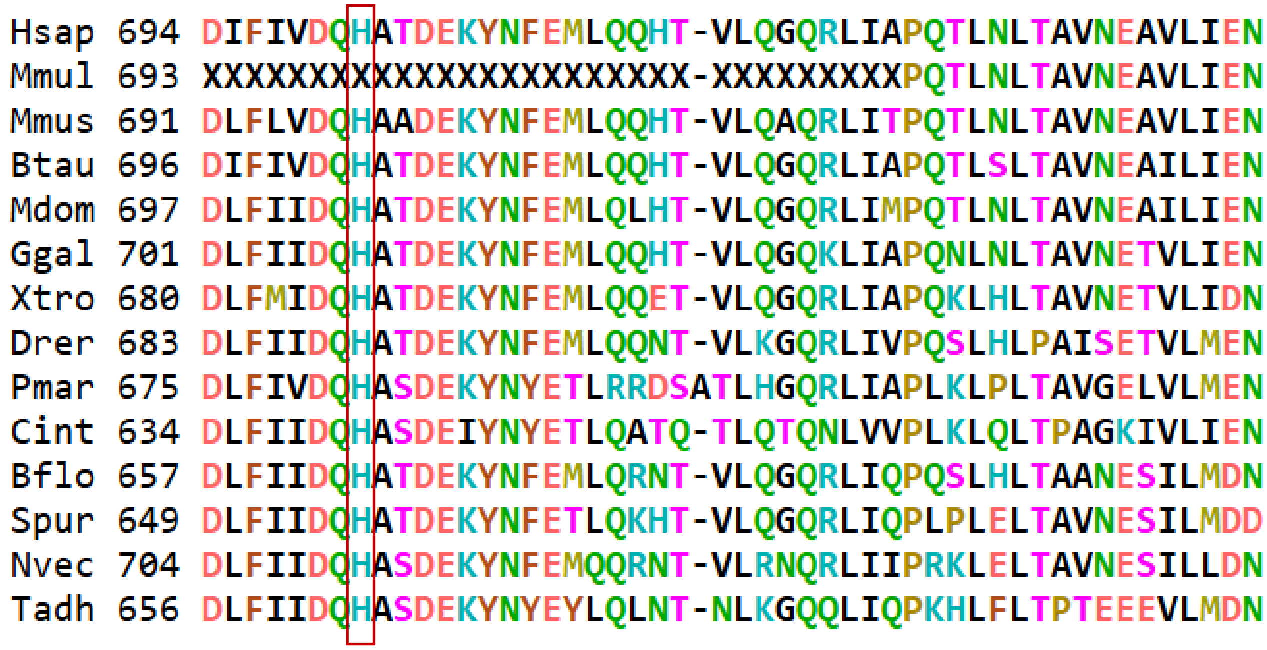

Aa Res	Aa Ref	Aa Sub	gv	gd	Prediction
701	H	Q	0	24.1	C15

Figure S1. Evolutionary conservation of the PMS2 His701 residue. Multi-sequence alignment illustrating the evolutionary conservation of the His701 residue across vertebrate and invertebrate species in the region encompassing the *PMS2* p.His701Asn missense variant identified in the studied patients. The alignment was generated using the Align-GVGD sequence library (http://agvgd.hci.utah.edu; accessed June 2026). Abbreviations: Hsap, *Homo sapiens*; Mmul, *Macaca mulatta*; Mmus, *Mus musculus*; Btau, *Bos taurus*; Mdom, *Monodelphis domestica*; Ggal, *Gallus gallus*; Xtro, *Xenopus tropicalis*; Drer, *Danio rerio*; Pmar, *Petromyzon marinus*; Cint, *Ciona intestinalis*; Bflo, *Branchiostoma floridae*; Spur, *Strongylocentrotus purpuratus*; Nvec, *Nematostella vectensis*; Tadh, *Trichoplax adhaerens*.

Table S1. *In silico* meta-analysis prediction. *In silico* meta-analysis to predict the effect of the PMS2 NM_000535.7: c.2103T>A p.His701Gln (p.H701Q) missense variant, along with the calculation of the consensus score using the "Consensus Binary score" criterion, which is the proportion of binarized predictions that all predictors agree upon out of the total predictions.

***In Silico* Prediction Tools (link and References)**	**Deleterious Score Cutoff**	**NM_000535.7: c.2103T>A p.His701Gln (p.H701Q)**	**Consensus Binary score**
AlphaMissense (https://alphamissense.hegelab.org/;[1])	≥ 0.5	0.998 (Pathogenic)	0.05
CADD (*https://cadd.gs.washington.edu/info*; [2])	≤ 15.0	13.7 (Benign)	0
ClinPred (*https://sites.google.com/site/clinpred/home?authuser=0*; [3])	>0.5	0.997(Deleterious)	0.05
DEOGEN 2 (*https://rostlab.org/services/deogen2*;[4])	≥ 0.5	0.53 (Deleterious)	0.05
E-SNP&GO (https://esnpsandgo.biocomp.unibo.it/; [5])	≥ 0.5	0.86 (Pathogenic)	0.05
EVE (*https://evemodel.org*;[6])	≥ 0.5	0.95 (Pathogenic)	0.05
FATHMM (*http://fathmm.biocompute.org.uk/*;[7])	≤ -1.5	-3.66 (Neutral)	0
LIST-S2 (http://list-s2.mpi-inf.mpg.de/;[8])	≥ 0.5	0.999 (Deleterious)	0.05
MetaIR (*https://github.com/marciaah/metalr*; [6])	≥ 0.5	0.679 (Deleterious)	0.05
MetaSVM (*https://github.com/jjh0925/metaSVM*,[9])	≥ 0.0	0.326 (Deleterious)	0.05
MutPred2 (*http://mutpred.mutdb.org/*;[10])	≥ 0.5	0.825 (Deleterious)	0.05
Mutation Taster (www.mutationtaster.org; [11])	≥ 0.5	0.86 (Deleterious)	0.05
Mutation Assessor (http://mutationassessor.org/r3/; [12])	>0.8	3.71(High Deleterious)	0.05
PANTHER (*http://www.pantherdb.org*;[13])	≥ 0.5	0.95 (Probably Damaging)	0.05
PolyPhen-2 (*https://genetics.bwh.harvard.edu/pph2/*; [14])	≥ 0.5	0.99 (Probably Damaging)	0.05
PROVEAN (*http://provean.jcvi.org*; [15])	≤-2.5	-6.71 (Deleterious)	0.05
REVEL (*https://sites.google.com/site/revelgenomics*;[16])	≥ 0.5	0.817 (Pathogenic)	0.05
SIFT (*https://siftdna.org/*; [17])	≥ 0.05	0.81 (Deleterious)	0.05
**Consensus score**			**0.88**
		**PATHOGENIC**	

Table S2. Densitometric analysis related to Figure 5b. Raw densitometry values for β-actin, GFP, PMS2, and MLH1, normalized values, and Fold Changes relative to MLH1 + PMS2 WT (shaded row) are reported separately for three independent experiments (Experiments 1–3).

Experiments related to Figure 5b	Sample	Raw densitometry (a.u.)				PMS2 normalized values			MLH1 normalized values		
		β-actin	GFP	PMS2	MLH1	PMS2/GFP	PMS2/GFP/β-actin	Fold Change	MLH1/GFP	MLH1/GFP/β-actin	Fold Change
Experiment 1	EMPTY	3,098,590	14,681,236	12,887	532,275	0.0009	2.83 × 10–10	0.0007	0.0362	1.17 × 10–8	0.0910
	MLH1	3,525,088	15,437,125	168,963	2,730,982	0.0111	3.10 × 10–9	0.0077	0.1769	5.02 × 10–8	0.3902
	PMS2 WT	3,027,600	13,500,084	1,796,592	227,104	0.1331	4.40 × 10–8	0.1090	0.0168	5.56 × 10–9	0.0432
	PMS2 H701Q	3,155,261	11,191,662	941,651	110,450	0.0841	2.67 × 10–8	0.0661	0.0099	3.13 × 10–9	0.0243
	PMS2 E705K	3,121,382	12,668,352	1,865,382	385,259	0.1472	4.72 × 10–8	0.1170	0.0304	9.74 × 10–9	0.0757
	MLH1 + PMS2 WT	3,256,267	15,967,536	20,965,119	6,687,980	1.3130	4.03 × 10–7	1.0000	0.4188	1.29 × 10–7	1.0000
	MLH1 + PMS2 H701Q	3,551,477	16,308,136	2,416,344	3,450,495	0.1482	4.17 × 10–8	0.1035	0.2116	5.96 × 10–8	0.4632
	MLH1 + PMS2 E705K	3,485,240	19,478,140	4,799,970	2,283,363	0.2464	7.07 × 10–8	0.1753	0.1172	3.36 × 10–8	0.2615
Experiment 2	EMPTY	3,504,978	8,709,168	309,288	162,288	0.0355	1.01 × 10–8	0.0287	0.0186	5.32 × 10–9	0.0800
	MLH1	4,292,181	9,669,744	765,429	1,242,983	0.0791	1.84 × 10–8	0.0523	0.1285	2.99 × 10–8	0.4509
	PMS2 WT	4,268,090	11,484,252	3,351,200	242,501	0.2918	6.84 × 10–8	0.1937	0.0211	4.95 × 10–9	0.0745
	PMS2 H701Q	4,473,037	11,916,850	2,391,004	225,694	0.2006	4.49 × 10–8	0.1271	0.0189	4.23 × 10–9	0.0637
	PMS2 E705K	5,292,280	10,869,600	4,038,433	197,274	0.3715	7.02 × 10–8	0.1989	0.0181	3.43 × 10–9	0.0516
	MLH1 + PMS2 WT	4,489,836	11,112,192	17,605,820	3,314,037	1.5840	3.53 × 10–7	1.0000	0.2982	6.64 × 10–8	1.0000
	MLH1+ PMS2 H701Q	3,190,360	11,069,350	4,353,750	1,808,180	0.3933	1.23 × 10–7	0.3494	0.1633	5.12 × 10–8	0.7708
	MLH1 + PMS2 E705K	2,840,304	10,082,394	6,334,719	1,476,160	0.6282	2.21 × 10–7	0.6268	0.1464	5.15 × 10–8	0.7760
Experiment 3	EMPTY	3,603,512	4,578,840	803,658	12,402	0.1755	4.87 × 10–8	0.0339	0.0027	7.52 × 10–10	0.0261
	MLH1	3,662,052	4,396,728	1,548,774	263,703	0.3523	9.62 × 10–8	0.0670	0.0599	1.64 × 10–8	0.5684
	PMS2 WT	3,623,500	4,629,520	6,339,922	8,268	1.3690	3.78 × 10–7	0.2633	0.0018	4.93 × 10–10	0.0171
	PMS2 H701Q	3,605,961	4,451,645	4,250,460	16,165	0.9548	2.65 × 10–7	0.1844	0.0036	1.01 × 10–9	0.0349
	PMS2 E705K	3,486,156	4,390,020	6,754,469	22,366	1.5386	4.41 × 10–7	0.3074	0.0051	1.46 × 10–9	0.0507
	MLH1 + PMS2 WT	3,335,920	4,591,836	21,990,345	441,378	4.7890	1.44 × 10–6	1.0000	0.0961	2.88 × 10–8	1.0000
	MLH1 + PMS2 H701Q	3,358,476	4,244,520	6,307,925	93,480	1.4861	4.43 × 10–7	0.3082	0.0220	6.56 × 10–9	0.2276
	MLH1 + PMS2 E705K	3,162,771	4,990,328	11,904,012	69,428	2.3854	7.54 × 10–7	0.5254	0.0139	4.40 × 10–9	0.1527

Raw densitometry values are expressed in arbitrary units (a.u.)

Table S3. Statistical analysis of fold-change values for Figure 5b. Two-tailed one-sample Student's *t*-test comparing Fold Change values of each sample from three independent experiments (Experiments 1–3) with the reference value of 1 from the MLH1 + PMS2 WT sample.

Sample	Fold Change Experiment 1	Fold Change Experiment 2	Fold Change Experiment 3	Fold Change Mean	Standard Deviation	t statistic value	Degrees of freedom	p (two-tailed)
**PMS2**								
EMPTY	0.0007	0.0287	0.0339	0.0211	0.0179	−94.88	2	0.0001
MLH1	0.0077	0.0523	0.0670	0.0423	0.0309	−53.72	2	0.0003
PMS2 WT	0.1090	0.1937	0.2633	0.1886	0.0772	−18.19	2	0.0030
PMS2 H701Q	0.0661	0.1271	0.1844	0.1259	0.0592	−25.59	2	0.0015
PMS2 E705K	0.1170	0.1989	0.3074	0.2078	0.0955	−14.36	2	0.0048
**MLH1 + PMS2 WT**	1.0000	1.0000	1.0000	1.0000	0.0000	*reference*		
MLH1 + PMS2 H701Q	0.1035	0.3494	0.3082	0.2537	0.1317	−9.81	2	0.0102
MLH1 + PMS2 E705K	0.1753	0.6268	0.5254	0.4425	0.2369	−4.07	2	0.0552
**MLH1**								
EMPTY	0.0910	0.0800	0.0261	0.0657	0.0347	−46.59	2	0.0005
MLH1	0.3902	0.4509	0.5684	0.4698	0.0906	−10.13	2	0.0096
PMS2 WT	0.0432	0.0745	0.0171	0.0449	0.0287	−57.58	2	0.0003
PMS2 H701Q	0.0243	0.0637	0.0349	0.0410	0.0204	−81.43	2	0.0001
PMS2 E705K	0.0757	0.0516	0.0507	0.0594	0.0142	−114.79	2	$7.59 \times 10{-}5$
**MLH1 + PMS2 WT**	1.0000	1.0000	1.0000	1.0000	0.0000	*reference*		
MLH1 + PMS2 H701Q	0.4632	0.7708	0.2276	0.4872	0.2724	−3.26	2	0.0826
MLH1 + PMS2 E705K	0.2615	0.7760	0.1527	0.3967	0.3330	−3.14	2	0.0883

Figure S2

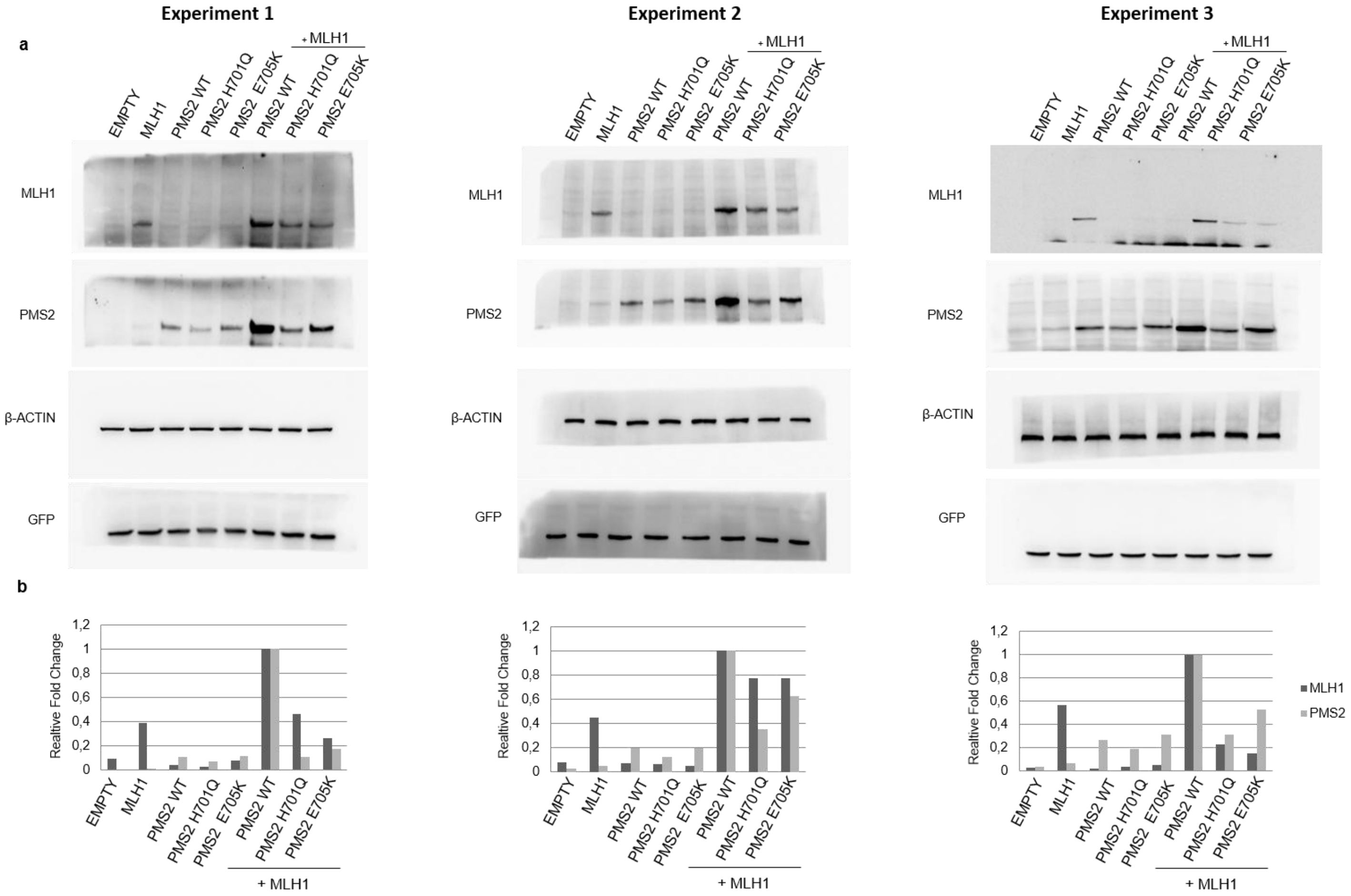

Figure S2. Western blot analysis from three independent experiments related to Figure 5a and 5b. (a) Uncropped original membrane image of three independent biological replicates. Immunoblot analysis of PMS2 and MLH1 in HEK-293T cells transiently transfected for 48 hours with pEGFP-N1 and pcDNA3.1 EMPTY or pSG5 hPMS2-WT, and either pcDNA3.1_hMLH1 wild-type (WT) or pSG5_hPMS2_H701Q or pSG5_hPMS2_E705K. β-actin was used as a loading control, and GFP served as a transfection control. (b) Densitometric analysis of the Western blot was performed by first normalizing MLH1 and PMS2 expression levels to GFP to assess transfection efficiency, and subsequently to the loading control (β-actin). Fold Changes relative to MLH1 + PMS2 WT were represented for each sample in three independent experiments (Experiments 1–3).

Table S4. Densitometric analysis related to Figure 5d. Raw densitometry values for MLH1 and PMS2 in the input and in the MLH1 immunoprecipitate (IP-MLH1), PMS2/MLH1 ratios, and Fold Changes relative to MLH1 + PMS2 WT are reported separately for three independent experiments (Experiments 1–3).

Experiments related to Figure 5d	Fraction	Lane	Raw densitometry (a.u.)		IP-MLH1 normalized values	
			MLH1	PMS2	PMS2/MLH1	Fold Change
Experiment 1	Input	EMPTY	75,335	3,168	—	—
		MLH1 + PMS2 WT	8,408,960	1,354,536	—	—
		MLH1 + PMS2 H701Q	2,945,410	239,598	—	—
		MLH1 + PMS2 E705K	3,018,340	523,799	—	—
	IP MLH1	EMPTY	51,935	2,304	—	—
		MLH1 + PMS2 WT	10,687,062	2,173,840	0.2034	1.0000
		MLH1 + PMS2 H701Q	3,471,390	301,785	0.0869	0.4273
		MLH1 + PMS2 E705K	3,122,864	474,525	0.1519	0.7470
		IgG MLH1	127,010	2,784	—	—
Experiment 2	Input	EMPTY	77,472	14,688	—	—
		MLH1 + PMS2 WT	729,840	849,744	—	—
		MLH1 + PMS2 H701Q	208,032	90,336	—	—
		MLH1 + PMS2 E705K	168,768	338,900	—	—
	IP MLH1	EMPTY	12,528	6,048	—	—
		MLH1 + PMS2 WT	695,084	505,056	0.7266	1.0000
		MLH1 + PMS2 H701Q	211,920	103,632	0.4890	0.6730
		MLH1 + PMS2 E705K	217,248	125,232	0.5764	0.7933
		IgG MLH1	75,072	2,880	—	—
Experiment 3	Input	EMPTY	97,900	21,573	—	—
		MLH1 + PMS2 WT	6,934,450	10,502,736	—	—
		MLH1 + PMS2 H701Q	2,130,150	2,757,432	—	—
		MLH1 + PMS2 E705K	1,673,728	2,920,965	—	—
	IP MLH1	EMPTY	134,850	111,639	—	—
		MLH1 + PMS2 WT	8,593,536	11,096,670	1.2913	1.0000
		MLH1 + PMS2 H701Q	2,882,300	1,748,347	0.6066	0.4697
		MLH1 + PMS2 E705K	3,171,700	2,335,062	0.7362	0.5701
		IgG MLH1	509,000	307,377	—	—

Raw densitometry values are expressed in arbitrary units (a.u.).

Table S5. Statistical analysis of fold-change values for Figure 5d. Two-tailed one-sample Student's t-tests comparing the PMS2/MLH1 (IP-MLH1) Fold Change of each sample from three independent experiments (Experiments 1–3) against the reference value of 1 from the MLH1 + PMS2 WT sample.

Sample	Fold Change Experiment 1	Fold Change Experiment 2	Fold Change Experiment 3	Fold Change Mean	Standard Deviation	t statistic value	Degrees of freedom	p (two-tailed)
**PMS2/MLH1 (IP-MLH1)**								
**MLH1 + PMS2 WT**	1.0000	1.0000	1.0000	1.0000	0.0000	*reference*		
MLH1 + PMS2 H701Q	0.4273	0.6730	0.4697	0.5234	0.1313	−6.29	2	0.0244
MLH1 + PMS2 E705K	0.7470	0.7933	0.5701	0.7035	0.1178	−4.36	2	0.0488

Figure S3

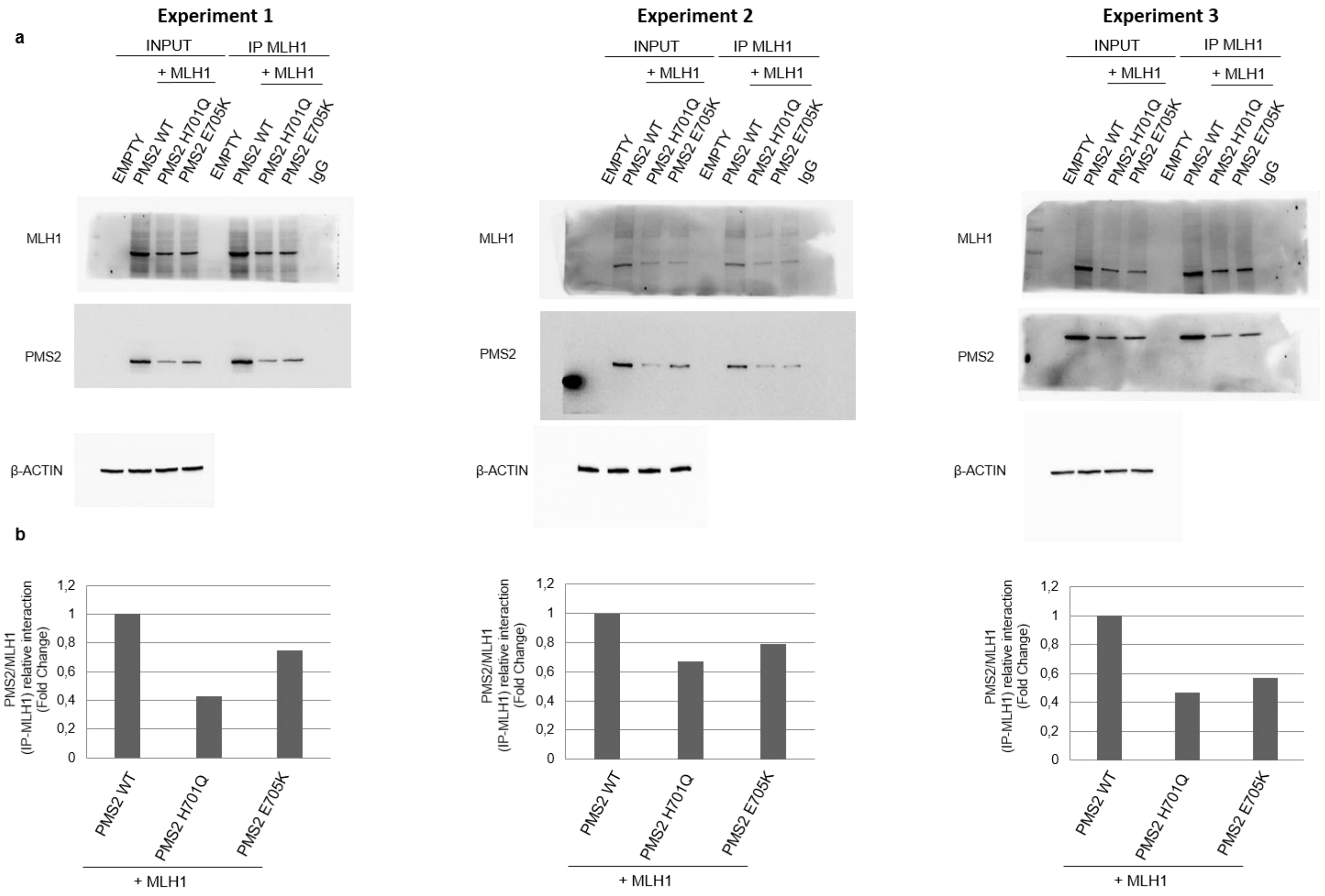

Figure S3. Co-IP analysis from three independent experiments related to Figure 5c and 5d. (a) Uncropped original membrane image of three independent biological replicates. Co-IP analysis of the MutLα complex in HEK-293T cells transiently transfected for 48 hours with pcDNA3.1 EMPTY, pcDNA3.1_hMLH1 wild-type (WT) and either pSG5 hPMS2-WT or pSG5_hPMS2_H701Q or pSG5_hPMS2_E705K. Immunoprecipitation was performed using an anti-MLH1 antibody, and IgG was used as a negative control. (b) Densitometric analysis of co-immunoprecipitation (co-IP) assays was performed by normalizing the PMS2 (prey) signal intensity to that of MLH1 (bait). PMS2/MLH1 (IP-MLH1) Fold Changes relative to MLH1 + PMS2 WT were represented for each sample in three independent experiments (Experiments 1–3).

Supplementary References

1. Nguyen, T.N.; Lee, T.; Turaga, N.; Gentleman, R.; Geistlinger, L.; Morgan, M. AlphaMissenseR: An Integrated Framework for Investigating Missense Mutations in Human Protein-Coding Genes. *Bioinform Adv* **2025**, *5*, vbaf093, doi:10.1093/bioadv/vbaf093.
2. Schubach, M.; Maass, T.; Nazaretyan, L.; Röner, S.; Kircher, M. CADD v1.7: Using Protein Language Models, Regulatory CNNs and Other Nucleotide-Level Scores to Improve Genome-Wide Variant Predictions. *Nucleic Acids Res* **2024**, *52*, D1143–D1154, doi:10.1093/nar/gkad989.
3. Alirezaie, N.; Kernohan, K.D.; Hartley, T.; Majewski, J.; Hocking, T.D. ClinPred: Prediction Tool to Identify Disease-Relevant Nonsynonymous Single-Nucleotide Variants. *Am J Hum Genet* **2018**, *103*, 474–483, doi:10.1016/j.ajhg.2018.08.005.
4. Raimondi, D.; Tanyalcin, I.; Ferté, J.; Gazzo, A.; Orlando, G.; Lenaerts, T.; Rooman, M.; Vranken, W. DEOGEN2: Prediction and Interactive Visualization of Single Amino Acid Variant Deleteriousness in Human Proteins. *Nucleic Acids Res* **2017**, *45*, W201–W206, doi:10.1093/nar/gkx390.
5. Manfredi, M.; Savojardo, C.; Martelli, P.L.; Casadio, R. E-SNPs&GO: Embedding of Protein Sequence and Function Improves the Annotation of Human Pathogenic Variants. *Bioinformatics* **2022**, *38*, 5168–5174, doi:10.1093/bioinformatics/btac678.
6. Dong, C.; Wei, P.; Jian, X.; Gibbs, R.; Boerwinkle, E.; Wang, K.; Liu, X. Comparison and Integration of Deleteriousness Prediction Methods for Nonsynonymous SNVs in Whole Exome Sequencing Studies. *Hum Mol Genet* **2015**, *24*, 2125–2137, doi:10.1093/hmg/ddu733.
7. Rogers, M.F.; Shihab, H.A.; Mort, M.; Cooper, D.N.; Gaunt, T.R.; Campbell, C. FATHMM-XF: Accurate Prediction of Pathogenic Point Mutations via Extended Features. *Bioinformatics* **2018**, *34*, 511–513, doi:10.1093/bioinformatics/btx536.
8. Malhis, N.; Jacobson, M.; Jones, S.J.M.; Gsponer, J. LIST-S2: Taxonomy Based Sorting of Deleterious Missense Mutations across Species. *Nucleic Acids Res* **2020**, *48*, W154–W161, doi:10.1093/nar/gkaa288.
9. Riesselman, A.J.; Ingraham, J.B.; Marks, D.S. Deep Generative Models of Genetic Variation Capture the Effects of Mutations. *Nat Methods* **2018**, *15*, 816–822, doi:10.1038/s41592-018-0138-4.
10. Pejaver, V.; Mooney, S.D.; Radivojac, P. Missense Variant Pathogenicity Predictors Generalize Well across a Range of Function-Specific Prediction Challenges. *Hum Mutat* **2017**, *38*, 1092–1108, doi:10.1002/humu.23258.
11. Steinhaus, R.; Proft, S.; Schuelke, M.; Cooper, D.N.; Schwarz, J.M.; Seelow, D. MutationTaster2021. *Nucleic Acids Res* **2021**, *49*, W446–W451, doi:10.1093/nar/gkab266.
12. Su, Y.; Li, X.; Reva, B.; Antipin, Y.; Schultz, N.; Bruijn, I. de; Sander, C. MutationAssessor in cBioPortal 2025, 2025.08.10.669566.
13. Thomas, P.D.; Campbell, M.J.; Kejariwal, A.; Mi, H.; Karlak, B.; Daverman, R.; Diemer, K.; Muruganujan, A.; Narechania, A. PANTHER: A Library of Protein Families and Subfamilies Indexed by Function. *Genome Res* **2003**, *13*, 2129–2141, doi:10.1101/gr.772403.
14. Adzhubei, I.A.; Schmidt, S.; Peshkin, L.; Ramensky, V.E.; Gerasimova, A.; Bork, P.; Kondrashov, A.S.; Sunyaev, S.R. A Method and Server for Predicting Damaging Missense Mutations. *Nat Methods* **2010**, *7*, 248–249, doi:10.1038/nmeth0410-248.
15. Choi, Y.; Sims, G.E.; Murphy, S.; Miller, J.R.; Chan, A.P. Predicting the Functional Effect of Amino Acid Substitutions and Indels. *PLoS One* **2012**, *7*, e46688, doi:10.1371/journal.pone.0046688.
16. Ioannidis, N.M.; Rothstein, J.H.; Pejaver, V.; Middha, S.; McDonnell, S.K.; Baheti, S.; Musolf, A.; Li, Q.; Holzinger, E.; Karyadi, D.; et al. REVEL: An Ensemble Method for Predicting the Pathogenicity of Rare Missense Variants. *Am J Hum Genet* **2016**, *99*, 877–885, doi:10.1016/j.ajhg.2016.08.016.
17. Vaser, R.; Adusumalli, S.; Leng, S.N.; Sikic, M.; Ng, P.C. SIFT Missense Predictions for Genomes. *Nat Protoc* **2016**, *11*, 1–9, doi:10.1038/nprot.2015.123.
